# Clinical implications of synovial tissue phenotypes in rheumatoid arthritis

**DOI:** 10.3389/fmed.2023.1093348

**Published:** 2024-05-22

**Authors:** Vivian P. Bykerk

**Affiliations:** Hospital for Special Surgery, New York, NY, United States

**Keywords:** synovial biopsy tissue, rheumatoid arthritis, RNA sequencing, immunohistochemistry, review

## Abstract

Autoimmune forms of inflammatory arthritis, such as Rheumatoid Arthritis (RA), are clinically heterogeneous in presentation and disease course. Treatment-related outcomes vary despite patient exposure to similar treatment strategies. It is likely that variation seen in synovial pathogenesis influences outcomes and is heterogeneous outcomes influenced by patient factors, including environmental exposures, microbiota, behaviors, timely access to therapy, and synovial cell variation. Patients’ unique complex factors manifest as specific synovial phenotypes characterized by clusters of synovial cell types and states. Precision medicine aims to use such clinical and biological data to identify the right treatment for the right patient at the right time, enabling patients to achieve sustained remission. Identifying synovial targets susceptible to a given treatment, enabling the choice of effective therapy for a given patient, will realize the goals of precision medicine. Over the last 7 years, improved acquisition and processing of synovial tissue obtained by ultra-sound guided biopsy has enabled researchers to define synovial pathotypes using histologic features and predominant cell types associated with clinical manifestations. Technical advances have enabled single-cell simultaneous sequencing of proteins and gene expression that, through increasingly sophisticated bioinformatics methods, have taken transcriptional and proteomic data to identify diverse and novel cell types and states that cluster in the RA synovium to further define patient subgroups. Synovial pathotypes and endotypes are now integrated into clinical studies and trials to explain clinical heterogeneity in disease course and treatment response. Rapidly evolving clinical-translational research has linked an expanded understanding of RA synovial pathogenesis with clinically meaningful subgroups and treatment outcomes and the clinical heterogeneity in RA.

## 1 Introduction

**Figure 1:**
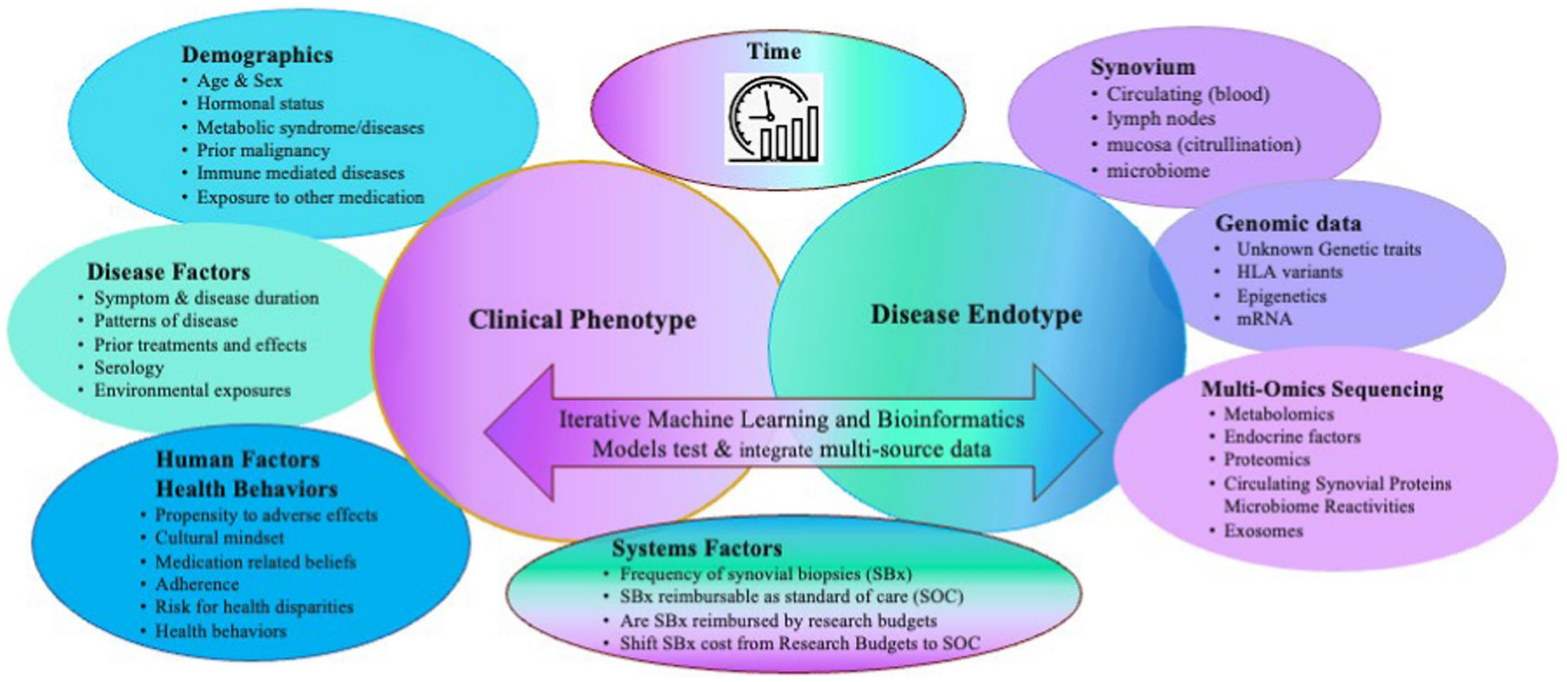
Multiple clinical and biological factors contribute to the heterogenous clinical and endotypic phenotypes of RA and need to be considered as covariates when generating predictive models of clinical outcomes. Adapted with permission Humby et al. (2019) (11), licensed under CC BY 4.0, https://doi.org/10.1136/annrheumdis-2018-214539.

Autoimmune forms of inflammatory arthritis, such as Rheumatoid Arthritis (RA), are clinically heterogeneous in presentation, disease course, and outcomes despite using similar treatment strategies. As a result, patients with RA and suboptimal outcomes have many unmet needs. Despite advances in targeted therapies, most patients with RA cannot achieve sustained remission, often flare, find their therapies become secondarily ineffective, or continue to lose function and experience progressive deformities—no cure for RA is on the horizon. Multiple factors may contribute to heterogeneity, including external exposures, a patient’s microbiota, and behavioral factors such as adherence and health behaviors, timely access to the most appropriate therapy, genomics, and epigenetics (Figure 1). Each may influence the expression of disease, which in RA coalesces in the synovium. Using clinical factors alone to predict disease course and response to treatment is imprecise. Biological factors may be better predictors. Precision medicine aims to inform treatment choices using clinical and biological data to predict the right treatment for the right patient at the right time, enabling patients to achieve sustained remission (1). One approach to achieve this is identifying synovial cell endotypes by upregulated molecular pathways likely susceptible to certain treatment targets and treating accordingly.

This narrative review will describe the recent evidence from clinical-translational research in RA that has associated clinical phenotypes with an increasingly deeper understanding of synovial tissue endotypes as they manifest, giving rise to RA pathogenesis. The characterization of synovial tissue by histology and immunohistochemistry, informed by transcriptional data from high throughput, simultaneous proteomic, and RNA sequencing of single cells from RA synovia, demonstrated never before identified cell types and states in the synovium of early and established RA that can be present despite the duration of disease or treatment exposures. Observational studies and trials have associated synovial tissue and clinical data to appreciate endotypes that are more or less likely to predict improvement in disease activity in patients with treatment-naïve early RA initiating methotrexate (MTX), to patients who have failed their first biologic therapy to predict what transcriptional profile and synovial cells might predict resistance to other mechanism of action, advanced therapies. These efforts are advancing the goals of precision medicine in which, based on clinical-biologic phenotypes, the right treatment can be chosen for the right patient at the right time. Choosing a therapy for patients with newly identified RA, those who have inadequate responses to a DMARD, or who have become treatment-resistant is challenging for all patients who live with this disease. At any point during the course of RA, unmet needs limit what patients can achieve, let alone sustain in terms of clinical and synovial remission.

Synovial patterns using histology and transcriptomic data can redefine RA phenotypes and predict clinical outcomes based on varying treatment exposures using machine learning-derived algorithms. These are being validated in additional studies for their utility in precision-based care. This has been possible because of broadening expertise in obtaining high-quality synovial tissue samples using ultrasound-guided synovial biopsies from large numbers of patients (2), expanded analyses of histopathology, and molecular sequencing of synovial cells through improved methods of synovial tissue disaggregation yielding larger numbers of live-sorted single cells for high throughput multiomic sequencing technologies. Classifying synovial pathology has moved from a scoring system using H&E-stained synovium and three synovial features only evaluated in arthroplasty tissue to an expanded number of synovial features present in inflammation, identifying cell subtypes using an expanded number of clinical differentiation (CD) markers via immunohistochemistry, mass spectrometry, and RNA sequencing and doing so in synovial biopsy tissue. Clinical-pathologic association studies link newly defined synovial classification systems, using molecular data with clinical features of inflammation to develop predictive algorithms of clinical phenotypes, treatment response, and disease activity.

In mechanistic synovial tissue research, efforts now involve applying high throughput sequencing technologies to disaggregated sorted synovial single cells to identify novel inflammatory cell types and states in RA patients’ synovial biopsies. Bioinformatic integration and methods identify novel cell types and states in RA synovial tissue from heterogeneous high-dimensional transcriptomic and proteomic data to re-classify synovial tissue, expanding our understanding of synovial pathotypes, which are then linked to clinical phenotypes and responses to therapy. This has set the stage to link novel synovial subtypes based on multiple cell populations with disease activity metrics in cross-sectional and longitudinal clinical studies and associate these with disease outcomes and treatment responses.

This review summarizes the research from studies linking clinical data synovial histopathology and transcriptionally informed synovial endotypes used to subgroup patients as well as results of translational comparative effectiveness randomized trials using transcriptomic informed cellular phenotypes to predict treatment-related clinical outcomes of remission and refractory disease.

## 2 Pathologic histological synovial phenotypes (pathotypes) and related scoring systems associated with clinical measures

### 2.1 Advances in classifying synovial tissue into subgroups or pathotypes were built on the original Krenn synovitis score, histologic features unique to RA, and dominance of cell types defined by cluster differentiation markers

Krenn et al. (3) first derived a semi-quantitative scoring system using arthroplasty-based synovial tissues from patients with inflammatory and degenerative arthritis. In the KSS, scores from three synovial features (range 0–3), including (i) lining layer thickness, (ii) inflammatory infiltrate, and (iii) stromal cell density, are summed for an overall score (range 0–9) reflecting the extent of synovitis. Scores ranging from 0 to 1 indicate no synovitis, 2 to 4 indicate low-grade synovitis and 5 to 9 indicate high-grade synovitis. A score of ≥5 is highly associated with an inflammatory joint disease (sensitivity of 62%, specificity of 96%). When applied to synovial biopsy tissue, the KSS can be a variable integrated with other clinical features into a simple predictive algorithm for response to methotrexate in RA.

However, there are limitations with the KSS when used specifically for RA research, as several features of inflammation are not accounted for. Carr and File noted the KSS did not account for synovial tissue organization and adapted the scoring system to incorporate infiltrates and the degree of organization of perivascular lymphoid aggregates, an indicator of active RA with poor prognosis. While using H&E-stained tissue, they included synovial infiltrate density and perivascular lymphoid aggregate size. They derived a four-point semiquantitative score using biopsies taken from treatment-naïve early RA patients. They added a four-point semi-quantitative scale (0–3) for cellular infiltrate density, aggregate size, and number according to a well-defined image atlas (4).

### 2.2 H&E synovial scoring applied to synovial biopsy tissue combined with clinical metrics can predict outcomes in treatment-naïve patients with early RA

Alivernini et al. (5) performed synovial biopsies in 545 patients with RA and followed these patients with clinical metrics for 6 months. Each tissue was graded using the KSS. They developed a nomogram that could predict which of the 245 patients with treatment-naïve RA could achieve DAS28 remission 6 months after initiating methotrexate (MTX) using three variables based on logistic regression analysis. Patients with very early RA (VERA, symptom duration of <3 months), a KSS score of <5, and a DAS28 score of <5.1 had an 82% probability of achieving remission. However, this nomogram has limited use, and additional synovial characterization is needed to predict outcomes of patients with RA across the spectrum of disease.

### 2.3 Synovial inflammation was further characterized using specific features identified on sections of RA synovium stained with H&E and predicted using a genomics-informed weighted scoring system that also correlates with clinical measures

A recent study by Orange and DiCarlo et al. (6) demonstrated that a weighted synovial inflammation score using specific synovial features observed in the histology of H&E-stained sections of arthroplasty-derived tissue could be derived using the input of bulk RNA sequencing and clinical features (CRP, ESR, RF, anti-CCP) to derive a weighted scoring system that could predict high, low, and non-inflammatory tissue that correlated with clinical manifestations of the disease. Expert pathologists identified 20 previously published histological features in H&E-stained surgically obtained synovial tissues from 129 patients in this study. Gene expression data were generated in a derivation cohort of 45 patients using bulk RNA sequencing (RNAseq) to identify which synovial features were associated with inflammatory RA based on upregulated inflammatory genes and pathways. Using machine learning methods, the top 50 differentially expressed genes (DEG) and gene signatures indicating a high inflammatory state were associated with the top 10 histological features indicating inflammation and correlated with clinical data (CCP, ESR, RF, and anti-CCP3 status). The 10 histologic features most frequent in high inflammatory synovia were then weighted to derive a scoring system that can predict which tissues are in a high or low inflammatory state vs. those that were non-inflamed allowing the identification of synovial features seen in H&E-stained tissue alone to be used to score synovium. A library showing these histologic features is available for others to use,^1^ as is the algorithm to score these features.^2^

### 2.4 Staining of synovial tissue sections with cluster differentiation markers by immunohistochemistry to identify cell types can characterize RA synovial pathology

To characterize RA-related synovial pathology on synovial tissue sections specifically, investigators expanded the KSS score (0–9). They used CD markers of immunologic cell types to inform which cell types are present in inflamed synovial tissues (7). Namn et al. (8) added an “IMSYC” extension score based on CD-marker-stained cellular infiltrates (0–4 for the degree of infiltrate of four cell types, for a maximum score of 16, based CD68 for macrophages, CD3 for T-cells, CD20 for B-cells, and CD31 for Endothelial Cells). Their extended score enables specific scoring of inflammatory joint disease, according to their atlas guiding semiquantitative scoring for each cell type. ROC curves indicated a cut point of ≥13.5 on the combined score discriminated inflammatory and non-inflammatory synovitis (sensitivity 72%, specificity 98%). When switching CD31 endothelial markers to CD15 for neutrophil staining, the KSS + extended score positively and moderately correlated with the CDAI (*r* = 0.65, *p* = <0.001, *r* = 0.68, *p* = <0.001) in 62 treated patients with RA regardless of joint pattern, seropositivity, and steroid use. Interestingly, in TNFα inhibitor (TNFi)-treated RA patients, T and B cell and neutrophil staining was reduced, but not CD68 staining. Moreover, the KSS outperformed the IMSYC extension sub-score in MTX treated, but not TNFi-patients [MTX (*r* = 0.86) vs. TNFi (*r* = 0.55)], indicating the two therapies differentially reduce cells in the synovium (9). This approach could be integrated into clinical research.

### 2.5 Establishing that synovial tissue is homogenous among synovial biopsies of different joints in the same patient

Older studies previously confirmed that synovial histology is similar between tissues from separate joints from the same patient. Whether systemic factors similarly affect synovitis qualitatively and quantitatively was only established recently. To address whether different joints similarly manifest synovial pathogenetic features based on systemic inflammation, Triaille et al. compared ultrasound-guided synovial biopsies from a large and a small joint in 10 patients and assessed local semiquantitative histology and quantified TCR-beta variable sequences between an individual’s blood and paired joints (10). Authors noted the degree of synovial hyperplasia, inflammatory infiltrates, and the proportion of CD3-positive T cells between was positively correlated for each individual’s paired joints. The extent of transcriptomic expression of RA-related molecular pathways, including those related to TCR signaling, T cell co-stimulation, response to TNFα, and T cell clonotype enrichment, was similar between joint pairs for each individual. Signatures were most comparable in patients whose transcriptomic profiles indicated the presence of germinal centers. Although this was a small study, these data suggest that T-cell-based synovial pathotypes and synovial characteristics based on systemic inflammation will be similar in the synovium from different joints in individuals with RA, further justifying that synovial tissue research can be employed using tissue from different joints in any given patient.

### 2.6 Synovial biopsy tissue-stained sections for H&E and CD markers for lymphoid, myeloid, and fibroid cell types to semi-quantitatively score and identify synovial pathotypes corresponding with clinical measures and transcriptomic data

This approach was first used by Humby et al. to classify synovial biopsy tissue from 144 patients with incident treatment naïve RA who were recruited into the multi-center, observational “Pathobiology of Early Arthritis Cohort” (PEAC) study that aimed to link synovial tissue data with clinical outcomes (11). Consecutively recruited participants (*n* = 144, symptom duration <12 months) underwent synovial biopsies before initiating conventional synthetic (cs) DMARDs and again at 6 months after treatment in a subset of patients. Clinical data, including disease activity measured by the DAS28 at baseline and 6 months, and radiographic damage progression were assessed 1 year after initiating csDMARD treatment (85% MTX). DAS28 scores and EULAR response criteria defined clinical disease activity and improvement.

**Figure 2:**
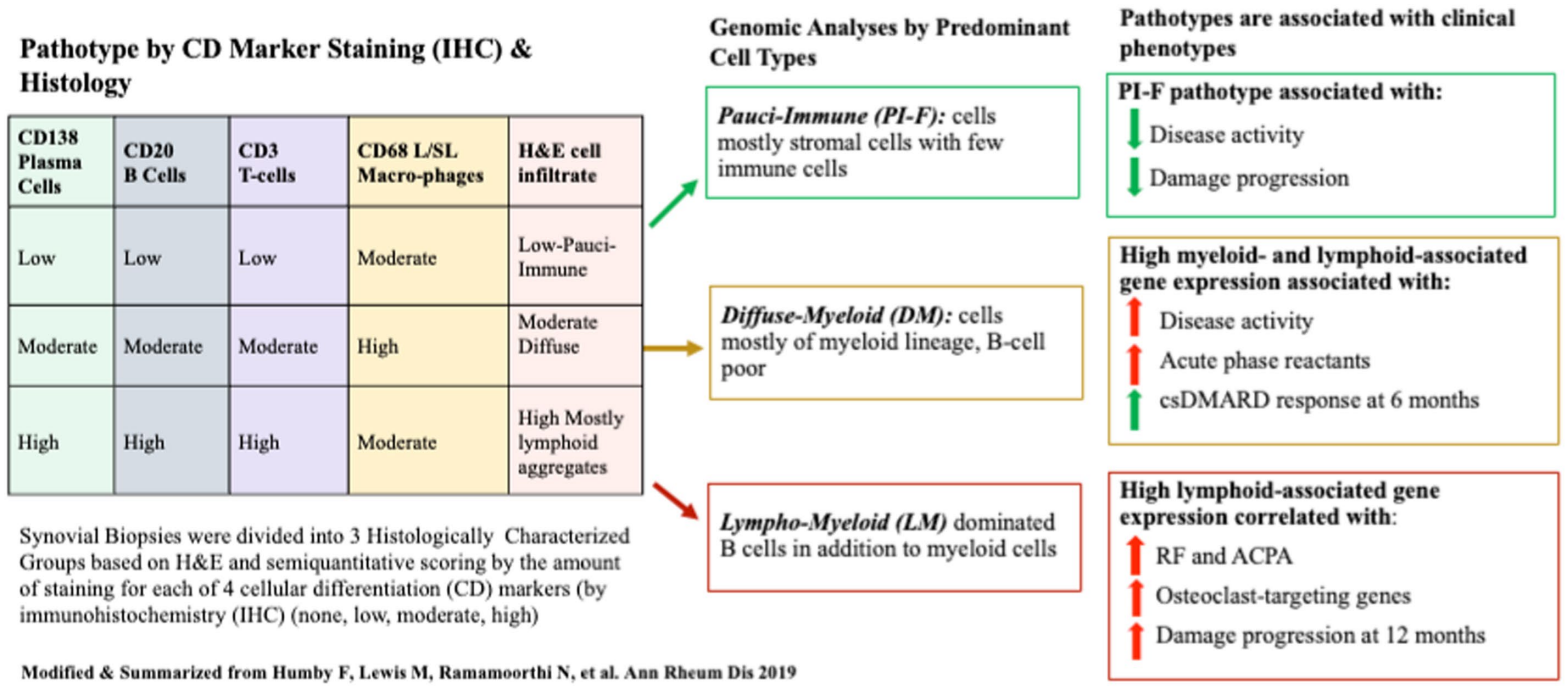
A schematic of the Pathobiology of Early Arthritis Cohort (PEAC) study of treatment-naïve patients with RA whose synovial tissues were semi-quantitatively scored according to four Cell Differentiation (CD) Markers to define three pathotypes at baseline. Pathotypes were associated with immune-cell lineage subtypes determined by gene expression data from bulk RNAseq. Pathotypes correlated with clinical metrics and disease activity outcomes 6 months after MTX/csDMARD initiation.

In their synovial tissue classification protocol, semiquantitative scoring and group classification were performed on tissues stained for H&E and CD20 for B-cells, CD3 for T-cells, CD138 for plasma cells, and CD68 for lining/sublining macrophages using IHC. Transcriptomic data were acquired for each sample using Nanostring® RNA sequencing. Each patient’s transcriptome cluster was assigned an eigengene score (EGS) reflecting the extent of expressed genes from lymphoid, myeloid, or fibroid cellular subsets. Histologic pathotype status and 46 differentially expressed genes (DEGs) were subsequently associated with clinical measures and outcomes. Patient samples clustered into three discrete histological pathotypes (1): *lympho-myeloid* (LM) being mostly B cells myeloid cells (2); *diffuse-myeloid* (DM) with myeloid lineage predominance (3) *pauci-immune-fibroid* (PI-F) with few immune cells and prevalent stromal cells (high EGS was high for fibroid cells). High Eigengene Scores (EGS) correlated with histologic-IHC-defined pathotypes in a 129-patient subset. Pathotypes were then related to clinical outcomes. Lymphoid- and Myeloid-EGS and LM and DM pathotypes were positively correlated with worse clinical metrics of RA at baseline but better clinical response to csDMARDs (mostly MTX) at 6 months. More damage progression was seen in the LM pathotype/lymphoid high EGS group at 1 year, but little was seen in the PI-F pathotype. Not surprisingly, more auto-antibodies were present in the LM pathotype/Lymphoid EGS, as were a higher number of osteoclast-targeting genes consistent with high damage. Additional positive correlations were seen between pretreatment serum levels of CXCL13 and MMP3 (but not sICAM1 and IL-8) levels, higher acute phase reactants, higher auto-antibody titers, and higher DAS28 and worse ultrasound scores with LM/DM pathotypes. The use of MTX reduced DAS28 the most in the LM/DM groups and less in the PI-F group. Despite clinical improvement in the LM/DM groups, most could not achieve low disease activity or remission at 6 months using csDMARDs as first-line treatment. A schema of the study and pathotype-related outcomes is shown in Figure 2.

A prediction model for developing damage progression at 1 year could be derived using eight clinical variables and 46 differentially expressed genes into a logistic regression model that could inform outcomes in over 85% of patients, excluding sequencing data. However, a model [including pathotypes, EGS (7 DEG), and RF] best-predicted outcomes [AUC 0.93 (95% CI 0.86–1.0)]. The predicted treatment response was higher based on DAS28, SJC, disease duration, and LM vs. DM or PI-F pathotypes. The LM pathotype had the highest weight for predicting damage progression. Thus, these IHC/H&E-based synovial tissue signatures in untreated RA are important in predicting treatment response and damage progression.

Importantly, a PI-F pathotype had not previously been appreciated in incident RA, yet 25% of patients with new-onset treatment naïve RA have pauci-immune synovium, showing not only fewer cells but also those present were predominantly fibroid and endothelial cells, with some myeloid elements. The implications of a pauci-immune pathotype on treatment response are important as some fibroblasts play a homeostatic role to support joint integrity and function, and others are pro-inflammatory and likely not targeted using currently available therapies for RA. Thus, further studies are needed to delineate specific cell states with a high abundance of highly active inflamed RA. Others have confirmed the utility of this approach, though they included markers of fibroblasts (CD90) and endothelial cells (CD31) to better distinguish between myeloid stromal and lymphoid pathotypes (12).

## 3 Advances in molecular and cellular phenotyping of synovial tissue in RA

### 3.1 Single-cell phenotyping by cellular markers and transcriptomics has revealed over 20 novel immune cell lineage subtypes and 77 activated cellular states to better understand RA pathogenesis, clinical response to treatment, and potential treatment targets

Several studies, mostly those by investigators collaborating with the NIH Accelerated Medicines Partnership for Rheumatoid Arthritis (AMP RA) (13), have developed standard methods and employed advanced technologies to identify novel cells in the synovium, including their cell states and function. Methods involved performing single-cell RNAseq, using Next-Generation sequencing (NGS), and using high numbers of cell-surface CD markers for cellular phenotyping by Mass cytometry using CyTOF. Molecular analyses were applied to ultrasound-guided synovial biopsy tissue fragments, allowing investigators to explore an exponentially increased number of differentially expressed genes (DEGs), pathways, and cell surface markers to characterize novel cell types and states. This first required developing a standardized protocol to disaggregate synovial tissue while preserving cell integrity and the ability to sort cells by CD markers into single-cell subsets using flow cytometry (14). As sequencing technologies advanced, the group worked to implement these using simultaneous multi-omic sequencing and expanded capacity to identify CD markers by CyTOF. New bioinformatic methods were needed to meaningfully reduce and harmonize exponentially increased multi-omic high-dimensional genomic data, including pathway analyses and CD markers in heterogeneous synovial tissues obtained from patients exposed to multiple therapies. They aimed to identify new cell types and functional states in RA inflammation and then subset this data to classify RA subtypes. Preliminary genomic and molecular data from earlier technologies were analyzed to cluster cell types and states and then associated with clinical and histologic characteristics to determine which could define meaningful subgroups (15). By concurrently applying advanced technologies to sorted cells from the same tissue samples, known and novel cell phenotypes based on surface CD marker data and upregulated genes associated with inflammatory pathways were identified in 25 cell subsets in RA tissues (16). Of these, 20 cell subsets could be related to inflammatory or homeostatic functional roles associated with histological synovial pathology. Supplementary Table 1 provides an overview of cell types, states, locations, and functions of known and novel T- and B-cells, fibroblasts, endothelial cells, and pro-inflammatory macrophage subsets based on these data and contemporaneous results from others incorporating new roles by cells promoting or inhibiting inflammation as a function of cell types, states and expressed genes and markers (17, 18).

**Figure 3:**
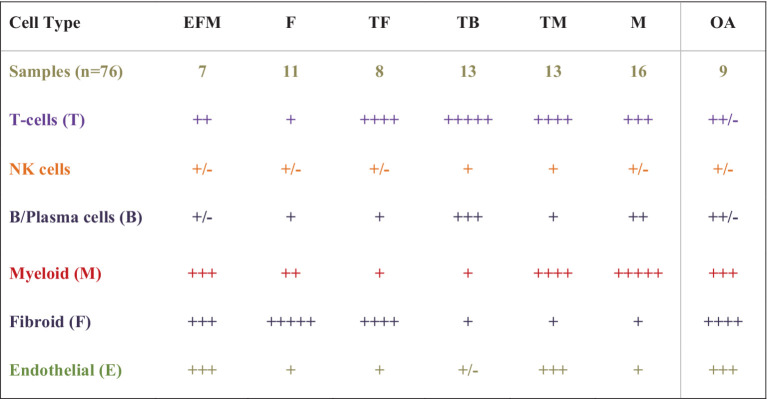
A schematic of cell dominant clusters generated using data from the AMP RA studies. Hierarchical clustering was used to reduce high-dimensional proteomic, scRNAseq, and Mass Cytometry data from 80 high-cell yield synovial tissues to identify six distinct groups by cell type abundance phenotypes (CTAPs), incorporating 18 unique cell subsets from five immune lineage cell types and over 70 cell states. Six RA CTAPs are labeled based on the relative proportions of cell types in each. Shown also are cell types in OA controls. Immune cell lineage subtypes included endothelial (E), fibroid (F), T- and B-cells (T and B), and Myeloid (M). Schematic depicts the proportion of cell type abundance: + < 5% ++ < 10%, +++ up to 25%, ++++ up to 50%, and +++++ up to 70% or more. RA patients were diverse with variable duration and treatment exposure (exp) (MTX none or low-exposed, MTX-exp or TNFi-exp) (Table is derived from NIH AMP-RA studies).

In the most recently completed AMP RA Phase II study aimed to deconstruct then reconstruct synovium into meaningful patterns, investigators combined multi-omic methods, [high throughput parallel 10X scRNA and proteomic sequencing (cite-seq) (CyTOF using 58 antibodies to define cellular CD markers)] to sequence genomic and proteomic data and concurrently cellular phenotypic data using high cell yield synovial biopsy samples (15). Bioinformatic synthesis of this high dimensional multiomic data from >328,000 FACs sorted single cells were combined with clinical and ultrasound data, histological parameters, using a newer consensus-based Birmingham-defined H&E histologic classification based on cell-density and organization were used to identify the most meaningful cell types and states present in the synovia of 69 RA patients and eight OA controls, confirming 18 cell types across six immune cell lineages, consistent with those previously identified [T-cells, NK-cells, stromal (fibroid) and myeloid cells, B-and Plasma-cells, and Endothelial Cells]. Expanded analyses demonstrated over 20 cell populations in 75 cell states to further understand their role and function in RA pathogenesis. In the reconstruction phase, hierarchical cluster analysis identified six unique cell type abundance phenotypes (CTAPs) from seven cell lineage subtypes and populations defined by cell state. These CTAPs (depicted in Figure 3) are now being further evaluated in clinical studies.

Additional studies aim to better understand how cells in the recently proposed CTAPs inter-relate in synovial microenvironments, including the active signaling pathways and their gradients that up and downregulate pro-inflammatory cells. Sequential studies can inform changes in architecture following treatment in the clinical context of prior disease activity, treatment responses, or resistance, providing insights into treatment mechanisms and potential druggable targets in RA (19). Such approaches are already being incorporated into clinical trials.

## 4 Relating clinical observations with synovial pathobiology can be accomplished by applying clinical hypotheses linked to mechanistic data

### 4.1 Association of obesity with gene expression of synovial tissue

The comparison of clinical with synovial phenotypes based on gene expression can provide insights into the heterogeneity of the disease course. Given that being overweight (or obese; BMI ≥25) reduces the probability of achieving disease control early in new onset RA (20), and that this may be more closely related to central obesity, often observed in metabolic syndrome (METS), Alivernini et al. set out to determine if synovial tissue can provide insights into how obesity and METS affect the RA clinical presentation, disease activity, and ability to respond to csDMARDs when compared to tissue from patients with a healthy weight. Indeed, more lymphoid aggregates, sublining inflammatory macrophages, and B-cell infiltrates (CD68+, CD21+, and CD20+) were present in synovial tissues from overweight vs. healthy weight patients (21). Although no synovial differences were seen in MTX inadequate responders (MTX-IR) for either weight group, there was more residual synovitis with a higher number of CD68+, CD20+, and lining/sublining CD3+ cells with more expressing CCL3 and MyD88 based on transcriptomic profiles in overweight vs. healthy weight patients who achieved clinical disease control and ultrasound (US) remission.

These findings highlight that being overweight/obese and having other features of METS may be an important clinical covariate in interpreting associations between clinical and mechanistic data, particularly given that METS is highly linked to declining levels of gonadal hormones, more so in women than men. These data suggest dysglycemia and hormonal factors might contribute to the immuno-pathogenesis of RA and synovial heterogeneity. These clinical factors associated with high RA disease activity may also be associated with upregulated inflammatory cells in states less susceptible to standard therapies.

### 4.2 Are patients in clinical remission also in histological remission, and if not, are the same genes expressed in the synovium as seen with active inflammation?

Orange et al. (22) recently assessed the synovia of RA patients with controlled disease defined as a DAS28 < 3.2, yet many still had subclinical synovitis (as measured on their histologic inflammation score). Of patients with controlled disease activity, either remission [DAS28 < 2.6 (14%)] or Low disease activity [LDA, DAS28 < 3.2(15%)], synovitis was still present in 27 and 31%, respectively. Patients in LDA with histological synovitis had higher C-reactive protein (CRP) levels (*p* = 0.0006) and higher anti-cyclic citrullinated peptide (anti-CCP) antibody levels (*p* = 0.03) compared to patients without subclinical synovitis. Of those with subclinical synovitis and controlled RA who also had available transcriptomic data, 183 differentially expressed genes associated with synovitis were still expressed. Thus, in arthroplasty patients with a DAS28 < 3.2, just under one-third still exhibit histologic evidence of subclinical synovitis, which could be associated with increased CRP and anti-CCP levels and will likely still have an expression of genes associated with inflammation in their synovium. These data argue for considering a histologic method of classifying synovitis when considering how long to keep patients off therapy around the time of joint surgery.

### 4.3 Are patients with histologically defined synovial pathotypes that are poor in B-cells less likely to respond to rituximab in a randomized clinical trial?

**Figure 4:**
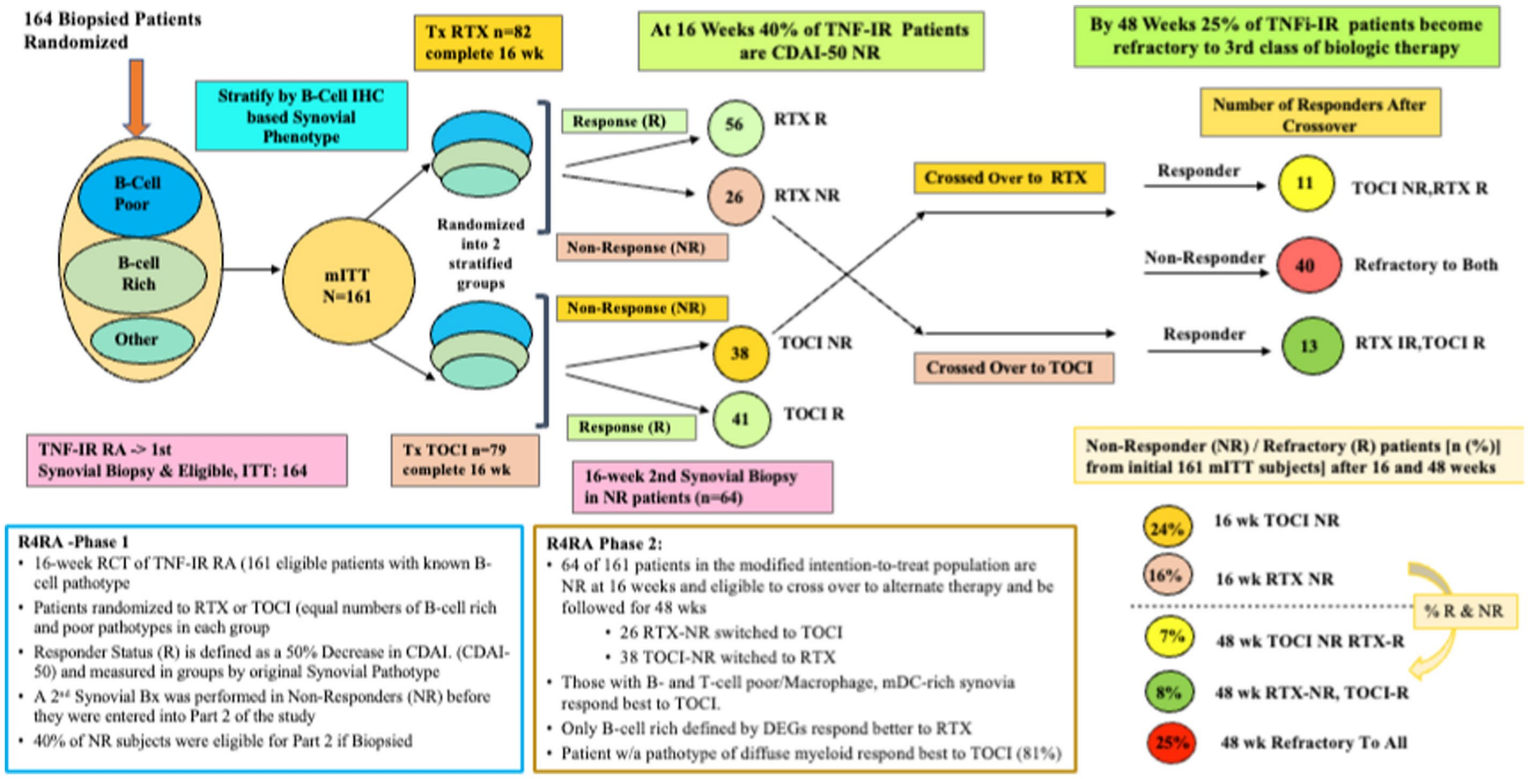
A schematic of the study design and outcome from both phases of the R4RA trial showing response status at 16 weeks in those randomized to Tocilizumab (TOCI) or Rituximab (RTX) and responses at 48 weeks in the non-responders subsequently crossed over to the alternate third biological therapy.

Whether histological classification of synovitis will suffice in predicting treatment outcomes is a question tackled in the R4RA study (23). The R4RA study was a 2-part, 48-week, biopsy-driven, multicenter, open-label randomized trial to test the hypothesis that patients with B-cell poor synovium on immunohistology would be less likely to respond to rituximab (RTX) vs. tocilizumab (TOCI). MTX-treated, TNF-IR patients with 9 years of RA (*N* = 161, age 55, 80% female, 88% seropositive, CDAI of 30) were recruited from five countries (19 centers in the United Kingdom, Belgium, Italy, Portugal, or Spain). Each underwent a baseline synovial biopsy and a repeat biopsy at 16 weeks if they were treatment-IR. Histology (w/IHC) of BL synovial biopsy—used to classify patients as B-cell poor or rich. They were randomized (1:1) to either RTX (2 × 1 g doses) or TOCI (8 mg/kg Q4wk), with equal proportions of B-cell poor and rich synovium in each treatment group. The synovial tissue characterization for the primary outcome used the histological staining and scoring protocol previously described in the PEAC study but adapted the scoring process to only score CD20 staining. In addition, sequencing data from patients’ synovial tissues were used, and B-cell rich/poor classification using genomic data from RNA-seq resulted in some patients’ synovia being reclassified based on the B-cell molecular signature in prespecified *post-hoc* analyses. The primary and secondary outcomes included a 50% improvement in CDAI from BL (CDAI50) at 16 weeks, but the secondary outcome also required patients to achieve Low Disease Activity (LDA) or remission. Figure 4 represents a schema depicting the 16- and 48-week study method and clinical outcomes of the R4RA study.

Despite a viable hypothesis, this group found no significant differences in CDAI50 response between treatments in patients with histologically characterized B-cell poor pathotypes at 16 weeks regardless of treatment arm; thus, based on the primary outcome, this was considered a failed study. However, more TOCI-treated (46%) vs. RTX-treated patients (24%) met the pre-specified, more stringent alternate outcome, namely achieving both a CDAI 50 + CDAI LDA (*p* = 0.035). Furthermore, when the B-Cell Poor phenotype was defined using RNA-seq data, significantly more TOCI-treated patients achieved both outcomes (CDAI50/CDAI50 + CDAI LDA) (63/50%) vs. RTX-treated patients (36/12%) (*p* = 0.035, *p* = 0.0012).

Patients (*n* = 53) who had failed to achieve a CDAI-50 were designated as non-responders (NR), underwent a second synovial biopsy, and were crossed over to receive the alternate therapy and followed for 48 weeks. The second phase patients included 26 RTX-NR patients (21%) who switched to TOCI and 38 TOCI-NR (32%) who switched to RTX (24). At the study’s end, one in three patients was refractory (R) to the second therapy and designated as RTX-R (9%) or TOCI-R (12%). Interestingly, refractory patients had synovia enriched with a stromal/fibroblast signature, including both pro-inflammatory and homeostatic (24).

Predictors of responders and refractory patients differed. Higher T-cell, B-cell, and Monocyte/Macrophage counts identified using RNAseq were associated with higher treatment response. In contrast, higher fibroblasts and stromal cells at 48 weeks following the crossover study were associated with being refractory to trials of both TOCI and RTX and TNF inhibitors. In addition, a machine learning algorithm was developed in a derivation set and refined in a test set that could distinguish between responders and non-responders using clinical, immunological, histology, and DEGs. The most stringent predictive model incorporating only expressed genes was most predictive of RTX and TOCI response (sensitivity AUC 0.83 and 0.86) and Refractory disease (0.71).

There are few transcriptomic change data associated with TNFi therapy. Recently, the level and effect of 12 weeks of newly initiated TNFi therapy on immune cell subsets and pathways were studied in a small cohort (*n* = 42) of patients with established RA, failing MTX, and/or other conventional DMARD (25). Bulk RNAseq data determined the proportionate number of DEGs upregulating immune cells and pathways pre-treatment. Then, numbers were downregulated in synovial biopsies taken before and 12 weeks after TNFi therapy. Changes in amounts of immune cells and pathways were estimated between clinical responders with a good ACR/EULAR response vs. moderate to poor responders. Fewer women were in the good response group, with less excess pain, lower CRP, and DAS28-CRP at baseline. Patients who were TNFi-good responders had greater elevations in immune pathways, including those responsible for chemokine signaling, antigen processing, and presentation, Fc gamma R-mediated phagocytosis, Th1 and Th2 cell differentiation, and Toll-like receptor as well as B- and T-cell receptor signaling that were all downregulated after therapy. Similarly, good responders had elevated immune cell lineage cells, including CCR7+ T cells, CD3+ Tph & Tfh cells, Tregs, CD8 GZMK+ T cells, Plasmablasts, CD19+ Memory and Age-associated B cells, IFN activated monocytes, C1QA monocytes, HLA-DRhi sublining, and lining fibroblasts were significantly reduced after 12-weeks of treatment. These data provide evidence for immune changes that can be induced by blocking TNF alpha in active RA. Still, more studies are needed to determine if similar changes might occur with other biological therapies. Also, there was too little data to determine if these mechanistic data could predict treatment outcomes.

## 5 Summary

To advance our abilities to predict the ideal treatment targets of RA during the disease course has the potential to increase the proportion of patients achieving sustained remission. Whether prediction models only need specifically expressed genes or additional clinical information requires multiple data sources to be generated in clinical studies and then integrated into prediction models. Whether or not clinical variables such as age, sex, disease duration, environmental exposures, number and types of comorbidities, non-rheumatic medication exposures, and the effects of clinical states such as metabolic syndrome will improve biologically based predictive models has not yet been tested. Moreover, social, and human factors affecting access and adherence must be overcome to implement precision medicine effectively based on the research described. Currently, the target tissue of interest most likely to inform disease pathogenesis is the synovium; identifying biologically predictive information from cells transmigrating in blood to or from lymph nodes and close sites of citrullination has yet to be explored to determine if a non-biopsy-based approach to precision medicine might be feasible. Much has been learned about cell types, states, and gene expression, but less has been published about the epigenetics of synovial inflammation. This could lead to further approaches to druggable targets. Relating transcriptional data and predictive models to clinical metrics and outcomes also requires further study for these lines of research to become relevant during patient care. Whether synovial biopsies and synovial tissue assays can become routinely available for clinical care in a cost-effective manner and whether or when synovial biopsies can become routine and reimbursed are additional hurdles to the envisioned implementation of precision medicine.

These findings would not be possible without the advances in next-generation sequencing technologies, and more remains to be learned about the pathophysiology and heterogeneity of RA. Many of the newly defined cell lineage subsets have been identified independently by several groups and are relatable to synovial tissue pathotypes and measurable signs of inflammation. Whether or not RA patients can be sub-classified based on histologic, transcriptomic “pathotypes” or cell type abundance phenotypes to choose therapies that will be associated with clinical improvement or identify those likely to be resistant to one therapy or another will ultimately need to be studied in head-to-head comparative effectiveness trials. It is only a matter of time before these multiple data sources can be integrated to predict disease trajectories from the outset.

Gravallese and Firestein (26) have recently summarized the clinical aspects, therapeutic targets, and critical steps involved in the pathogenesis of RA. Pathogenic steps begin with antigen presentation by dendritic cells, macrophages, and B cells to T-cells, which become activated and differentiate into cytokine producers. Cytokines activate and drive immune synovial effector cells, particularly synovial fibroblasts, monocytes, and macrophages, which secrete proteins mediating joint and systemic pathology. Plasma cells and additional antigens generated by neutrophil extracellular traps (NETs) continue to amplify and sustain this loop (26). Although many advanced therapies target several steps in the immunopathologic process, effector cells are often not targeted. Identifying the key cellular drivers in each person to appreciate what targeted therapy will likely impact synovial neighborhoods is needed to prevent early disease from becoming chronic and intervene in refractory established disease. Whether this requires novel or combined targeted therapies to have the greatest impact on an RA subgroup characterized by their cellular phenotype requires additional research.

The next steps for collaborative consortia will be to employ synovial spatial transcriptomic methods to model synovial cell function and cellular crosstalk in geo-locations within the synovium. This can inform how pathologic cell groups evolve and revert and which combination of cell types must be targeted to stop their pro-inflammatory/destructive and amplifying effects. Such research could highlight how pathologic cells can be removed and replaced by homeostatic cells to sustain joints. The patient phenotype comprising demographics, disease severity, duration, prior exposures, and multimorbidity contributes to RA disease heterogeneity, creating a barrier to a simple cure for RA. However, employing multi-modal mechanistic and clinical research strategies to find ways to tip the synovium back to a state of clinical homeostasis may overcome the devastating physical and systemic impact of Rheumatoid Arthritis from its earliest stages.

## Author contributions

The author confirms being the sole contributor of this work and has approved it for publication.

## Footnotes

^1^ https://www.hss.edu/pathology-synovitis.asp

^2^ https://predictrasubtypes.shinyapps.io/shiny/
