# Supplementary material for: Clinical implications of synovial tissue phenotypes in rheumatoid arthritis
Source: Front Med (Lausanne). 2024 May 22;10:1093348. doi: 10.3389/fmed.2023.1093348 (PMC11150787; doi:10.3389/fmed.2023.1093348)
Supplement: Supplementary file 1 [file Table_1.pdf]

Supplementary Table 1 – Cell Types and States and Their Roles in RA Synovium

Lineage/CD Markers	Activated States	Location in Synovium	Inflammatory vs. Homeostatic	Primary Role & Function	Origin	Histologic, Pathotype, CTAP Associations	Therapeutic Targets or Response Data
**Fibroid: Fibroblasts CD45-, PDPN+**	CD34+	Sublining	Inflammatory	Recruit monocytes, Express IL6, CXCL12, CCL2	Innate & Infiltrates	Pauci-Immune, F containing CTAPs	
	MHC II (SC-F2, THY1+CD34-HLA-DRhi	Sublining	Inflammatory	MCHII antigen presentation; express cytokines to recruit leucocytes, Secrete IL-6, MMPs	Transformed FLS, highly expanded in Inflamed RA Synovium		Anti-IL6, Possibly by TNFi
	CD55+	Lining	Homeostasis	Support synovial fluid	Innate FLS		
	DKK3	Sublining	Homeostasis	Protect bone, Produce & Express CAMD1, COL8A2	Innate FLS		
	DFGRA+/PRG4+ Lubricin+	Sublining	Homeostasis	Synovial Homeostasis, support cartilage	Innate FLS		
**Endothelial Cells CD31+ CD146+**	Arterial (PODXL + NOTCH4+)	Sublining	Inflammatory	NOTCH 3 signaling, upregulate sublining fibroblast, transforms FLS to pro-inflammatory fibroblasts	Endothelial progenitor cells	Fibroid cells; Pauci-immune-F Pathotypes; F, EFM CTAPs	
**Myeloid: Monocytes/ Macrophages CD45+ CD14+**	CX3CR1+	Lining	Homeostatic	Express genes for immune-regulation, clear apoptotic cells, barrier formation		Myeloid Pathotype, M containing CTAPs	May be upregulated after abatacept therapy
	M1 monocyte subsets	Lining or Infiltrate	Inflammatory	Main producers of TNF-alpha; Depend on Local cytokines and chemokines	Inflammatory Monocyte Activation States		May respond to MTX, Targeted by TNFi, IL-6i, JAKi
	HBEGF+	Alongside fibroblasts	Inflammatory	Express NR4A3, PLAUR and CXCL2, Growth Factor HBEGF	Innate		NA
	MerTK-CD206-	Alongside fibroblasts	Inflammatory	Transform FLS to Inflammatory State, IFN-activated; Induce fibroblasts to secrete MMP1/3, RANKL, IL6 CXCL8, CCL2, CCL20			Possibly TNFi and anti-IL6 Therapies
**Lymphoid T-Cells CD45+ CD3+**	CD4+ T-cell	Lymphoid Tissue, Infiltrate synovium, form aggregates	Inflammatory		Bone Marrow via thymus, to Lymph Tissue, Circulate, Infiltrate Synovium	Lymphoid Pathotypes, T CTAPs	NA
	CCR7+ T-cell effector memory cells		Inflammatory	Antigen recognition			
	FOXP3+ Tregs		Anti-inflammatory, Homeostasis	Immune cell regulation			Downregulated by TNFi
	PD1hiCXCR5-Tph cells	Circulating and in lymphoid aggregates	Inflammatory	Support B-cell differentiation, antibody production, upregulate SLAM 5, expanded in seropos RA	Lymphoid Aggregates		Downregulated by TNFi
	CD4+HLA-DR+CD27-		Inflammatory	Cytotoxic Memory function	Lymphoid, Aggregates		Downregulated by TNFi
**Granzyme expressing CD8+ cells**	GZMK+	Leucocyte infiltrates	Inflammatory	CD helper role, Cytotoxic		Lymphoid Pathotypes, T - CTAPs	NA
	GNLY+GZMB+		Inflammatory	CD helper role, Cytotoxic			IL-6, TNFi, JAKi
	GNLY+GZMB+ CTLA4+		Inflammatory	CD helper role, Cytotoxic			IL-6, TNFi, JAKi
**B-Cells CD45+CD3-CD19**					Lymphoid, Aggregates	Lymphoid Pathotypes, B - CTAPs	
	IGHD+ CD27+ naïve	Lymphoid infiltrates					Not affected by TNFi therapy
	IGHG3+ CD270-memory		Inflammatory				Downregulated by TNFi
	ABC cells		Inflammatory				Downregulated by TNFi
**Plasmablasts**	IgG+, XBP1+	High in inflamed synovium	Inflammatory	IFNδ stimulation and autoimmunity	Lymphocytic Infiltrates	Not captured	Possibly by TNFi
**Plasma Cells**	CD16+CD56+ bright		Inflammatory	IgG+ and IgA+	Transformed	Not captured	NA
	CD16-CD56dim						NA
